# Improving genomic prediction for plant disease using environmental covariates

**DOI:** 10.1186/s13007-025-01418-0

**Published:** 2025-08-20

**Authors:** Charlotte Brault, Emily J. Conley, Andrew C. Read, Andrew J. Green, Karl D. Glover, Jason P. Cook, Harsimardeep S. Gill, Jason D. Fiedler, James A. Anderson

**Affiliations:** 1 Department of Agronomy and Plant Genetics, University of Minnesota, St. Paul, MN 55108 USA; 2 USDA-ARS, Plant Science Research Unit, St. Paul, MN USA; 3 Department of Plant Sciences, North Dakota State University, Fargo, ND USA; 4 Agronomy, Horticulture, and Plant Science Department, South Dakota State University, Brookings, SD USA; 5 Plant Sciences and Plant Pathology Department, Montana State University, Bozeman, MT 59717 USA; 6 USDA-ARS Cereal Crops Improvement Research Unit, Edward T. Schafer Agricultural Research Center, Fargo, ND USA

**Keywords:** Genotype-by-environment interaction, Plant breeding, Disease resistance, Genomic prediction, Wheat

## Abstract

**Background:**

Fusarium Head Blight (FHB) is a destructive fungal disease affecting wheat and barley, leading to significant yield losses and reduced grain quality. Susceptibility to FHB is influenced by genetic factors, environmental conditions, and genotype-by-environment interactions (GxE), making it challenging to predict disease resistance across diverse environments. This study investigates GxE in a long-term spring wheat multi-environment uniform nursery trial focusing on the evaluation of resistant lines in northern US breeding programs.

**Results:**

Traditionally, GxE has been analyzed as a reaction norm over an environment index. Here, we computed the environment index as a linear combination of environmental covariables specific to each environment, and we derived an environment relationship matrix. Three methods were compared, all aimed at predicting untested genotypes in untested environments: the widely used Finlay-Wilkinson regression (FW), the joint-genomic regression analysis (JGRA) method, and mixed models incorporating an environmental covariates matrix. These were benchmarked against a baseline genomic selection model (GS) without environmental covariates. Predictive abilities were assessed within and across environments. The results revealed that the JGRA marker effect method was more accurate than GS in within- and across-environment predictions, although the differences were small. The predictive ability slightly decreased when the target environment was less related to the training environments. Mixed models performed similarly to JGRA within-environment, but JGRA outperformed the other methods for across-environment predictions. Additionally, JGRA identified significant genetic markers associated with baseline FHB resistance and environmental sensitivity. Furthermore, location-specific genomic estimated breeding values were predicted, providing insights into genotype stability across varying locations.

**Conclusion:**

These findings highlight the value of incorporating environmental covariates to increase predictive ability and improve the selection of resistant genotypes for diverse, untested environments. By leveraging this approach, breeders can effectively exploit GxE interactions to improve disease management at no additional cost.

**Supplementary Information:**

The online version contains supplementary material available at 10.1186/s13007-025-01418-0.

## Background

Food staple production is facing multiple challenges: an increasing world population, unpredictable weather events, and the need to decrease pesticide use. To address these challenges, the selection of adapted varieties is critical. In the northern United States Great Plains, hard red spring wheat (*Triticum aestivum* L.) is the 3rd largest crop, after corn and soybean [59]. A very threatening disease in this region is Fusarium head blight (FHB), a fungal disease caused primarily by *Fusarium graminearum* [44]. It develops mostly during anthesis and causes bleached or shriveled kernels, as well as deoxynivalenol (DON) mycotoxin accumulation in the grains. High DON concentrations are harmful to humans and animals, making grain unmarketable. Quantitative trait loci (QTLs) of moderate to small effect have been identified and introgressed from Chinese wheat, especially from ‘SUMAI 3’ [2]. Further studies verified several QTL such as *Fhb1*, *Fhb3*, and *Fhb5* [2, 8, 62]. Nevertheless, FHB resistance remains a complex trait, with many small-effect loci involved [40, 50, 61]. The climate and the environment are known to strongly influence FHB susceptibility and DON accumulation in the grain [40, 51, 52].

Since 1995, resistance to FHB has been evaluated in the uniform regional scab nursery (URSN). Approximately 30 varieties are tested annually in inoculated trials at four to six locations. Across 30 years, 817 lines have been tested in 11 locations, encompassing Minnesota (MN), North Dakota (ND), South Dakota (SD) states, and for some years, Manitoba or Saskatchewan provinces (MB and SK, respectively) in Canada. The cultivars mostly originate from public breeding programs from the University of Minnesota (UMN), North Dakota State University (NDSU), South Dakota State University (SDSU), and Montana State University (MSU), with some contributions from private companies for some years. This multi-environment trial (MET) allows for a good characterization of FHB resistance across environments.

In the last two decades, genomic selection [4, 39] has become increasingly popular in plant and animal breeding [14]. It consists of using genomic information to train a genomic selection model with phenotypic data so that the prediction of test lines can be done using genomic data only [39]. The practical implementation of genomic prediction (GP) in breeding programs requires some genetic relatedness between the training set and the test set, good marker coverage, and a sufficient training set size [1, 21, 23, 25]. With enough resources, GP has proven to be efficient in improving genetic gain in many crops, including wheat [3, 13, 14, 36, 63].

However, GP is usually based on the entry-mean value across environments, or predictions are made within environments, thus ignoring the environmental effect and the interaction between the genotype and the environment [9].

In quantitative genetics theory, phenotypic variation is determined by genetics, environment, and genotype-by-environment (GxE) variances [18]. When the GxE variance is high, the ranking of genotypes changes across environments, which impacts the accuracy of breeding decisions. Indeed, allelic effects might not be consistent across environments [42, 56]. Thus, including GxE in the breeding pipeline can increase the accuracy of GP [9] and enable the selection of genotypes adapted to specific environmental conditions to be targeted [28].

One of the earliest methods of characterizing GxE variation involves regressing the phenotypic value against the mean phenotypic value for each environment and genotype as a reaction norm [16, 34, 65]. This method is now known as the Finlay-Wilkinson (FW) regression [19]. The regressor then contains an estimate of the environment’s suitability, and the genotype slope provides an estimation of its stability. This method has recently been adapted to incorporate environment covariates (ECs), making it possible to predict performance in an unknown environment [46, 58].

More recently, ECs have become more accessible for a wide range of locations and years and have been integrated into GP models using kernels [10–12, 24, 28, 41, 47]. In some cases, the ECs were used to compute an environmental relationship matrix (ERM) [10, 28, 43] in the same way as the genomic relationship matrix (GRM) to capture the covariance between untested environments. However, this method implies that all ECs have the same impact on the phenotypes. Lopez-Cruz et al. [35] regressed each EC against the phenotype in a mixed model to decompose the GxE into each environmental component. That requires a multiple testing procedure with many parameters to be estimated. To overcome that, Li et al. [31–33] searched for the most appropriate EC and associated time window through the critical environment regressor with informed search (CERIS) joint genomic regression analysis (JGRA) This method allowed them to characterize more closely the allelic effect of known genes on the flowering time trait [32]. Factor analytics is another method for overcoming the dimension problem of the prediction when considering the phenotypes in multiple environments as multiple traits [26, 49].

Using an environment index is a simple, univariate method to integrate GxE, however, the definition of a single variable integrating the environment effect is challenging. A synthetic variable from ECs can also be computed via various methods [46, 48, 57].

In recent years, multiple methods have been developed to predict GxE, but comprehensive comparisons between those methods are lacking. This study aims to provide a thorough comparison of GxE methods for predicting untested environments and untested genotypes using a multiyear wheat dataset applied to FHB-related trait prediction. Specifically, we tested whether including GxE in prediction models increases the predictive ability and which model is the most appropriate for this inclusion. In addition to the prediction, the significance of the markers for explaining the baseline resistance or sensitivity to the environment was characterized. Finally, the ranking of genotypes in each location was estimated. To the best of our knowledge, this is the first study to include ECs to predict GxE for disease traits, despite some evidence that disease-related traits are affected by GxE [55].

## Method

### Experimental design

**Fig. 1:**
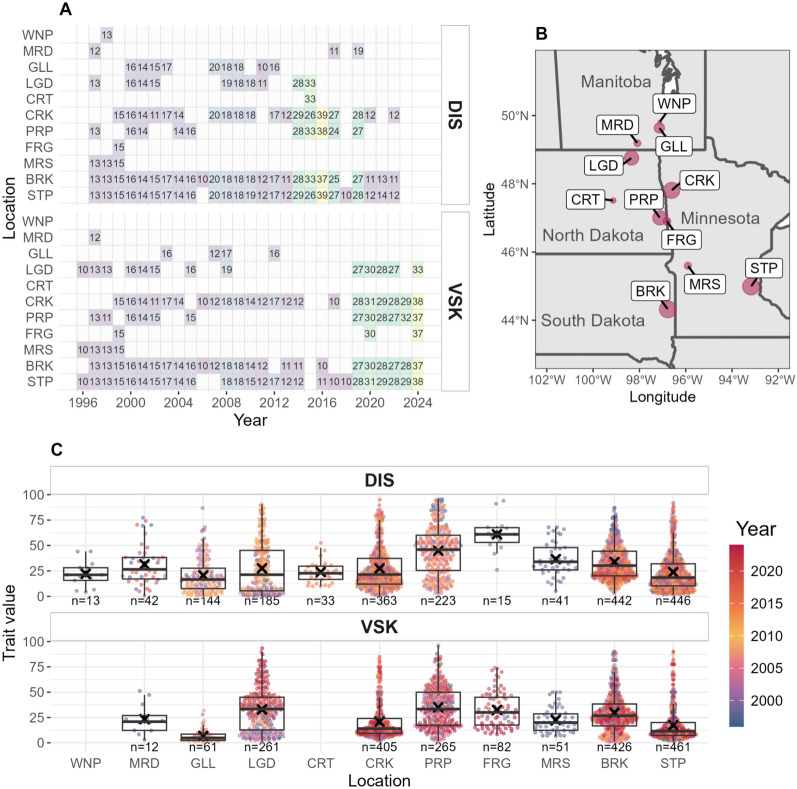
Description of the experimental design. **A** Experimental design for the selected lines, for disease index (DIS) and visual scabby kernel (VSK) traits. Each tile’s color represents the number of genotypes tested in that environment. **B** Map of the uniform regional scab nursery (URSN) locations. The size of the point indicates the number of times a location has been used in the 1995–2024 period. **C** Distribution of aggregated phenotypic data per location, colored by year of trial. The cross corresponds to the mean in each location, while the boxplot displays the quartiles. For both plots, the x-axis represents the locations of the uniform regional scab nursery (URSN) trials; the number of observations is indicated below each boxplot

The experimental design of the URSN population was described previously [5]. A randomized complete block design was used at each location with three blocks. After each year, the data were aggregated at the genotype level, accounting for field heterogeneity or outliers if necessary. Each year, three to six locations were used, spanning Minnesota, North Dakota, South Dakota, and Manitoba (Canada) for some years (Fig. 1A, B).

FHB phenotyping was performed for two traits: visual scabby kernel (VSK, expressed in percentage, crop ontology code CO_321:0001155) and disease index (DIS, expressed in percentage, crop ontology code CO_321:0501030), which is the multiplication between incidence and severity. The locations were inoculated with infected grain spawn, except for St. Paul (MN), for which the inoculation was done with inoculum spraying, and Brookings (SD), for which both methods were used. In the protocols, plots were inoculated around the anthesis date and misted to ensure good disease propagation.

The check ‘WHEATON’ was consistently used over the 30 years of experiments. Other checks have been used for many years, ensuring good connectivity.

From the initial dataset, individuals without genotypic data or tested in less than four environments were filtered out, and environments with fewer than 10 individuals were removed. The remaining dataset comprised 2,024 and 1,947 observations with 280 and 283 lines for 107 and 109 environments for VSK and DIS, respectively (Fig. 1A). Most of the time, genotypes were tested at all the locations for a given year.

Since no genotyping information was needed, phenotypic data from all available environments with more than 10 genotypes (n = 121 for DIS and n = 125 for VSK) were used for the determination of the environment index.

An analysis of variance was conducted after a first subset of the data to retain the genotypes tested for more than one year, leading to 83 genotypes remaining. A mixed effect model was fitted, such as: ${y}_{ijk}= \mu +{g}_{i}+{L}_{j}+{Y}_{k}+{(g:L)}_{ij}+ {(g:Y)}_{ik}+{(L:Y)}_{jk}+ {\epsilon }_{ijk}$, with ${g}_{i}$ the main genetic effect (83 levels), ${L}_{j}$ the location effect (11 levels), ${Y}_{k}$ the year effect (30 levels), and their corresponding interaction (:). All effects were fitted as random with independent and identically distributed variances.

### Genotypic data analysis

The lines were genotyped with the USDA-3K genotypic array, comprising 3421 single-nucleotide polymorphisms (SNPs), mapped on the IWGSC Chinese Spring reference genome v2.1 [67]. Markers and individuals with more than 25% missing data were excluded. Beagle software was used to impute the remaining missing data with default parameters [6, 7]. A minor allele frequency cutoff of 5% was used, leading to 2301 SNPs remaining for 467 individuals. The genomic matrix was scaled, and the additive genomic relationship matrix (GRM) was computed using the rrBLUP R package [17] following VanRaden [60] method as $G =M{M}^{\prime}/(2 {\sum }_{i=1}^{m}{p}_{i}(1-{p}_{i}))$, with ${M}_{ij}= {X}_{ij}+1-2{p}_{i}$, ${X}_{ij}$ being the genotype information for the line $j$ and the marker $i$, coded as 0, 1, or 2 for the number of alleles in common with the reference genome, ${p}_{i}$ the major allele frequency, and $m$ the number of markers.

### Environmental data analysis

The environmental data was extracted and combined from two sources: NASA (National Aeronautics and Space Administration) and NOAA (National Oceanic and Atmospheric Administration) weather stations (see Table S1 for the identification of the stations used). The following variables were used from NOAA stations: daily rainfall precipitation, minimum, maximum, and average daily temperatures. Furthermore, the R package *EnvRType*, through *nasapower*, was applied to search for the remaining ECs and to impute missing values based on the geographical coordinates of the field trials [10, 53, 54]. Other variables from the *EnvRtype* include computed variables related to plant growth and ecophysiology, such as potential evapotranspiration or the slope of the saturation vapor pressure curve, for example. The *soilType* R package was used to get 10 soil variables [20] based on the World Soil Database (WoSIS). A full description of the ECs is available in Table 1.

**Table 1:** Description of environmental covariates (ECs) obtained with *nasapower* and NOAA weather stations

Variable	Description	Unit	Location variable
ALLSKY_SFC_LW_DWN	All sky insolation incidence on a horizontal surface	MJ.m^-2^.d^-1^	FALSE
ALLSKY_SFC_PAR_TOT	All Sky Surface Photosynthetically Active Radiation (PAR) Total	W.m^-2^	FALSE
ALLSKY_SFC_SW_DNI	All Sky Surface Shortwave Downward Direct Normal Irradiance	W.m^-2^	FALSE
ALLSKY_SFC_SW_DWN	Thermal infrared longwave radiative flux	MJ.m^-2^.d^-1^	FALSE
ALLSKY_SFC_UVA	All Sky Surface Ultraviolet A (315 nm-400 nm) Irradiance	W.m^-2^	FALSE
ALLSKY_SFC_UVB	All Sky Surface Ultraviolet B (280 nm-315 nm) Irradiance	W.m^-2^	FALSE
ETP	Potential evapotranspiration	mm.d^-1^	FALSE
EVPTRNS	Evapotranspiration energy flux at the surface of the earth	MJ.m^-2^.d^-1^	FALSE
FROST_DAYS	If it was a frost day (temperature less than 0C or 32F)	-	FALSE
FRUE	Effect of temperature on radiation-use-efficiency	-	FALSE
GWETROOT	Root Zone Soil Wetness (layer from 0 to 100 cm), ranging from 0 to 1 (water-free to saturated soil)	-	FALSE
GWETTOP	Surface Soil Wetness (layer from 0 to 5 cm), ranging from 0 to 1 (water-free to saturated soil)	-	FALSE
LAT	Latitude	°	TRUE
LON	Longitude	°	TRUE
N	Photoperiod computed from latitude and Julian day	h	TRUE
n	Number of sunny hours in the day for a clear sky (no clouds)	h	FALSE
PAR_TEMP	Computed ratio between ALLSKY_SFC_PAR_TOT and T2M	-	FALSE
P-ETP	Computed difference between preciptation and evapotranspiration	mm.d^-1^	FALSE
PRECTOT	Rainfall precipitation	mm.d^-1^	FALSE
QV2M	Specific humidity at 2 m	%	FALSE
RH2M	Relative air humidity at 2 m	%	FALSE
RTA	Global solar radiation based on latitude and Julian day	W.m^-2^	FALSE
SNOW	Snow fall	mm	FALSE
SNWD	Snow depth	mm	FALSE
SPV	Slope of saturation vapor pressure curve	kPA.C°.d^-1^	FALSE
T2M	Temperature at 2 m	C°.d^-1^	FALSE
T2M_RANGE	Temperature range	C°.d^-1^	FALSE
T2MDEW	Dew-point temperature at 2 m	C°.d^-1^	FALSE
T2M_MAX	Maximum air temperature at 2 m	C°.d^-1^	FALSE
T2M_MIN	Minimum air temperature at 2 m	C°.d^-1^	FALSE
TH1	Temperature-Humidity Index by NRC 1971	C°.d^-1^	FALSE
TH2	Temperature-Humidity Index by Yousef 1985	C°.d^-1^	FALSE
VPD	Vapor pressure deficit	kPA.d^-1^	FALSE
WS2M	Wind speed at 10 m	m.s^-1^	FALSE
CLAY	Clay total	g/100 g	TRUE
CECPH7	Cation exchange capacity at pH7	Cmol(c)/kg	TRUE
ECEC	Effective cation exchange capacity	Cmol(c)/kg	TRUE
NITKJD	Total nitrogen	g/kg	TRUE
ORGC	Organic carbon	g/kg	TRUE
SILT	Silt total	g/100 g	TRUE
TCEQ	Calcium carbonate capacity	Cmol /kg	TRUE
WG0006	Water retention gravimetric 6 kPa	g/100 g	TRUE
WV0006	Water retention volumetric 6 kPa	Cm3/100cm3	TRUE
PHPTOT	Total phosphorus	mg/kg	TRUE
SAND	Total sand	g/100 g	TRUE
TOTC	Total carbon	g/100 g	TRUE

For each phenotyping environment, the daily environmental data were subset to dates between planting and harvesting. Finally, as in Costa-Neto et al. [10], we aggregated ECs across five wheat stages based on growing degree days, following Zadok’s scale [66].

Wheat stages and their corresponding cumulative growing Celsius degree-days since sowing (GDD):

- Stage 0–3 (V03) sowing to double-ridge: 400 GDD
- Stage 3–5 (V35) double-ridge to terminal spikelet: 685 GDD
- Stage 5–6 (V67) terminal spikelet to heading: 993 GDD
- Stage 6–7 (V67) heading to anthesis: 1075 GDD
- Stage 7–9 (V79) anthesis to maturity: 1825 GDD.

These values were obtained from the https://ipad.fas.usda.gov/cropexplorer/description.aspx?legendid=314 website (last accessed on 2024-03-17) and corrected for the heading date based on field observations.

After aggregation, the environmental data (W-matrix) comprised k = 143 ECs (26 weather variables divided into five stages, 10 soil ECs, and three location-specific ECs: latitude, longitude, and daylight hours) for 330 environments (for the 11 locations and each of the 30 years). Each column of the W-matrix was centered and scaled.

From the W-matrix, the environmental relationship matrix was computed following [10] as $\Omega =\frac{WW^{\prime}}{tr(W)/k}$, where $tr(W)$ is the trace of the W matrix and $k$ represents the number of ECs. The environments were characterized by their ECs through the ERM and Principal Component Analysis (PCA).

### Determination of the environment index

**Fig. 2:**
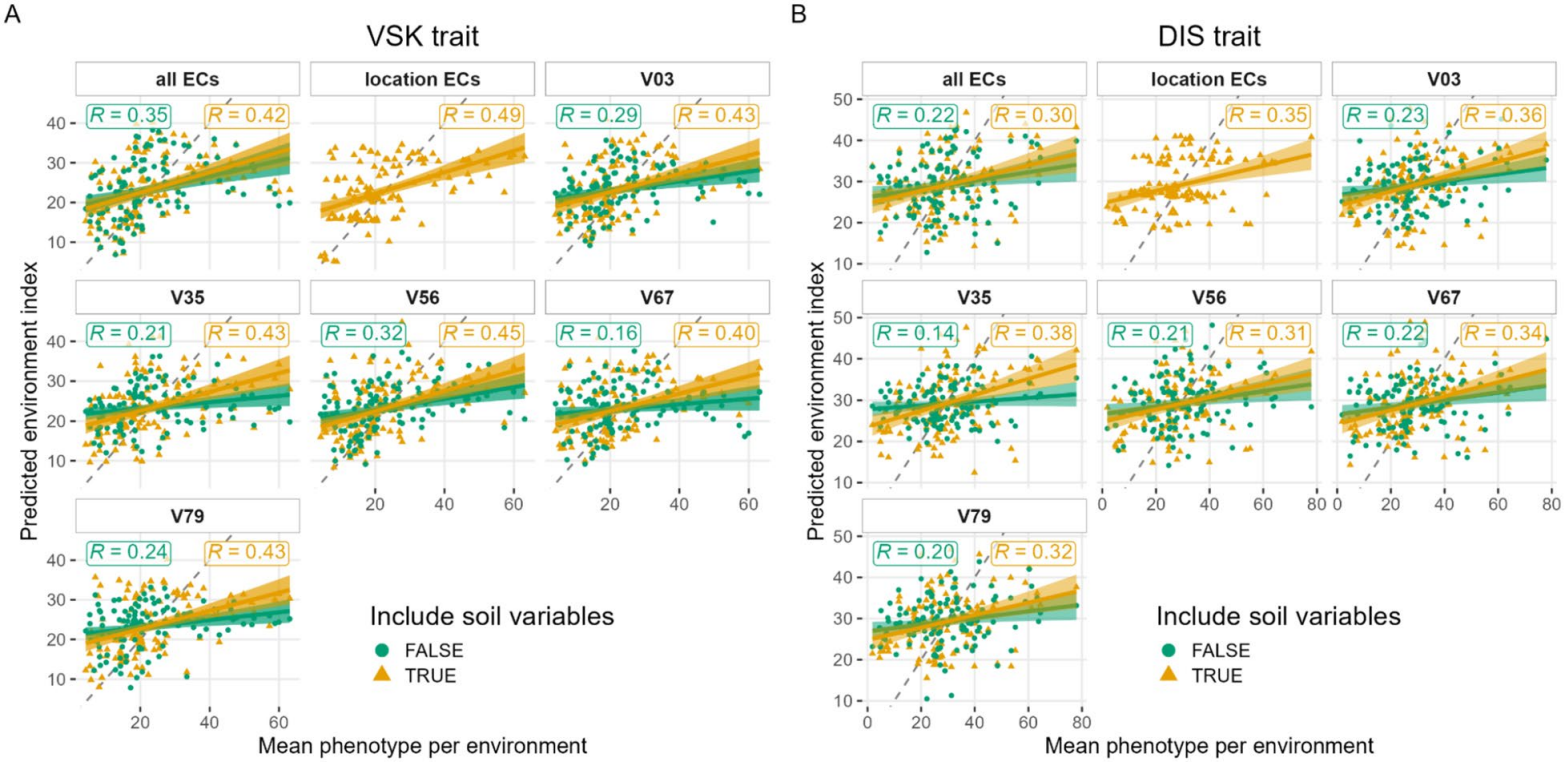
Determination of the environment index. Correlation between mean phenotype and predicted environment index for each environment, using all or a selection of environmental covariates (ECs). **A** for visual scabby kernel (VSK) trait, **B** for disease index (DIS) trait. The ECs specific to each location, encompassing soil variables, latitude, longitude, and daylight hours, were removed in one of the methods (green circle). Description of stages: V03: sowing to double-ridge, V35: double-ridge to terminal spikelet, V56: terminal spikelet to heading, V67: heading to anthesis, V79: anthesis to maturity

The environment index was computed as a linear regression between the ECs, with the relative weights predicted by partial least square regression (PLSR). This method is expected to account for the correlation between the ECs. The explanatory variable was the mean phenotype in each of the 121 or 125 environments, and the dependent variables were the ECs at different stages. Thirteen different sets of ECs were compared: fitting variables from the five wheat stages separately; all wheat stages, including or excluding location-specific variables (i.e., the 10 soil variables and latitude, longitude, and number of daylight hours), and soil variables only. Using a leave-one-environment-out cross-validation scheme for each trait, the PLSR method was applied for each combination of variables (the number of components was selected by an inner cross-validation using the Q2 parameter) and the Pearson correlation coefficient between predicted and observed mean phenotype was calculated (Fig. 2).

Some specific weather variables such as temperature, precipitation, and relative humidity are known to strongly affect FHB, especially around anthesis [29, 64]. To test that hypothesis, we computed the sum of those three variables averaged over ± 14 days around anthesis, determined as 993 GDD after sowing (De Wolf, personal communication).

### GxE methods

#### FW method

The R package FW [30] was used to compute the random regression of the phenotype against the mean environmental phenotype. First, intercept $\widehat{\mu }$ and slope $\widehat{\beta }$ parameters were estimated for each genotype using the FW method, using a Bayesian regression. The phenotypic data used here were phenotypic values obtained in each environment and beforehand corrected for spatial heterogeneity, subset for training set genotypes and training set environments. The intercept and slope parameters were subsequently predicted for the testing set genotypes by genomic prediction, using the ridge regression BLUP (RR-BLUP) method, using genomic markers. The final phenotypic value for the test set was determined via the PLSR environment index. This method was compared with JGRA as it corresponds to the most popular method, adapted here for prediction in an unknown environment.

#### JGRA

- **JGRA RN (reaction norm)**

In JGRA RN, for each line, a linear regression model is fitted for each genotype such as ${{\varvec{y}}}_{{\varvec{j}}}={\mu }_{j}+\delta {{\varvec{\beta}}}_{{\varvec{j}}}+{{\varvec{\varepsilon}}}_{j}$, with ${y}_{j}$ the ($p \times 1)$ vector of the phenotypic value for the genotype $j$ tested in several environments, ${\mu }_{j}$ and ${\beta }_{j}$ are the random genotype-specific intercept and the slope, respectively, $\delta$ is the vector of environment index for each environment (dimension $p \times 1$), and ${{\varvec{\varepsilon}}}_{j}$ the residuals. We assume that $\mu_j$_j_$\beta_j$_j_, and ${{\varvec{\varepsilon}}}_{j}$ are normally distributed, with a mean of 0 and with ${{\sigma }^{2}}_{\mu } {{\sigma }^{2}}_{\beta } {{\sigma }^{2}}_{\epsilon }$ variances. The normality of the residuals of that linear regression was assessed with a Shapiro–Wilk test, with 92% of the prediction combinations having a p-value superior to 0.05. The intercept and slope parameters were combined for all genotypes in the training set.

Then, intercept and slope parameters were predicted for the validation set by GP using RR-BLUP method and genomic markers. Finally, genotypic values were predicted as ${\widehat{{\varvec{y}}}}_{{\varvec{i}},{\varvec{j}}}= \widehat{{\mu }_{j}}+ {\delta }_{i}\widehat{{{\varvec{\beta}}}_{{\varvec{j}}}}$ for each genotype in each environment.

- **JGRA Marker Effect**

In the JGRA Marker Effect method, a GP model is fitted within each environment, such as ${{\varvec{y}}}_{{\varvec{i}}}=Z{{\varvec{u}}}_{{\varvec{i}}}+{{\varvec{\varepsilon}}}_{{\varvec{i}}}$, with ${{\varvec{y}}}_{{\varvec{i}}}$ the vector of phenotype for genotypes tested in the environment $i$, $Z$ is the marker matrix, $u$ is the vector marker effects, ${\varvec{u}}\sim N\left(0,I{\sigma }_{u}^{2}\right)$, with ${\sigma }_{u}^{2}$ the genetic variance, ${{\varvec{\varepsilon}}}_{{\varvec{i}}}$ and the residuals in that environment $i$. The normality of the residuals has been checked, for each scenario, trait and testing environment, 76% and 77% these combinations had residuals with a Shapiro–Wilk test p-value superior to 0.05 for DIS and VSK traits, respectively. Marker effects from all environments were combined. Then, for each marker, their effects were regressed against the environment index so that the marker effects could be predicted for a new environment based on the environment index. Then, the predicted phenotypic value for an unknown genotype and environment becomes: $\widehat{{{\varvec{y}}}_{{\varvec{j}}}}= \alpha + Z\widehat{{{\varvec{u}}}_{{\varvec{j}}}}$, with $\widehat{{{\varvec{y}}}_{{\varvec{j}}}}$ the predicted phenotypic value for the testing set genotypes in testing environment *j*, α the overall intercept, and $\widehat{{{\varvec{u}}}_{{\varvec{j}}}}$ the predicted marker effect for the environment *j*.

For comparison, genomic selection using the RR-BLUP method was also implemented, using only genomic data and without any ECs, with the same training and testing set partition as for the JGRA method.

#### Bayesian mixed models

Following Jarquín et al. [28], we compared different prediction models, including a combination of genotype (G), environment (E), and genotype-by-environment (GxE) effects.

- **Model G**

In that model, the genotype effect was included in the model, such as: ${\varvec{y}} = 1\mu + X{\varvec{\beta}}+{\varvec{\varepsilon}}$, with ${\varvec{y}}$ the vector of phenotypes in several environments of dimension $o\times 1$, $o$ being the number of observations, $\mu$ the overall mean, $1$ a vector of 1’s of dimension $o\times 1$, $X={Z }_{G}\times M$, with ${Z }_{G}$ the design matrix that connects the observation to the genotypes of dimension $o\times n$, with $n$ the number of genotypes, $M$ the scaled SNP matrix of dimension $n \times m$, with $m$ the number of SNP markers, and ${\varvec{\beta}}$ the unknown vector of random marker effects, of dimension $p\times 1$, and ${\varvec{\varepsilon}}$ the vector of residuals, assumed independent, identically distributed, and following a normal distribution with a variance of ${\sigma }_{\varepsilon }^{2}$. The effects were predicted using 10,000 burn-ins and 200,000 iterations, with the BGLR R package [45].

- **Model G + E**

In that model, the environment effect was added, such as: ${\varvec{y}}=1\mu +X{\varvec{\beta}}+S{\varvec{\gamma}}+{\varvec{\varepsilon}}$. $\text{S}={Z}_{E}\times \text{W}$, ${Z }_{E}$ is the design matrix that connects the observations to the environments of dimension $o\times t$ with $t$ the number of environments, $W$ is the matrix of ECs in each environment (dimension $t\times k$), and ${\varvec{\gamma}}$ is the unknown random environment effects.

- **Model G + GxE**

In that model, the interaction between the genotype and the environment was added, and the effects were predicted with the reproducible kernel Hilbert space (RKHS) method using a linear kernel, following Crossa et al. [15] and with the G effect as previously described. The G + GxE model is defined as: ${\varvec{y}}=1\mu +X{\varvec{\beta}}+gw+{\varvec{\varepsilon}}$, with $gw$ being the interaction between the genotype and the environment, with $gw\sim N\left(0,\left[({Z}_{G}G{{Z}^{\prime}}_{G})^\circ ({Z}_{E}\Omega {{Z}^{\prime}}_{E})\right]{{\sigma }^{2}}_{gw}\right)$, $^\circ$ denotes the Hadamard product between the matrices, $G$ and $\Omega$ are the genomic and the environmental relationship matrices, and ${{\sigma }^{2}}_{gw}$ is the variance due to GxE.

- **Model G + E + GxE**

In that model, all effects were included, such as ${\varvec{y}}=1\mu +X{\varvec{\beta}}+S{\varvec{\gamma}}+gw+{\varvec{\varepsilon}}$, with all the effects previously described.

The four models (G, G + E, G + GxE, and G + E + GxE) were compared with different methods: Bayesian ridge regression (BRR), Bayesian Lasso (BL), BayesA, BayesB, BayesC, and RKHS for fitting the genotype and the environment effects, while RKHS was constantly used to fit the GxE effect. For the RKHS method, the models were changed to accommodate for incorporating of the kernels corresponding to each of the effects. The BayesB method was retained and used across all four models because it provided the highest predictive ability on average (data not shown). In BayesB, a small proportion SNPs have non-zero effects and their variance follow an inverse chi-square distribution [39].

### Prediction scenarios

Several prediction scenarios were compared, all for predicting untested (unknown) genotypes by GP. The different scenarios tested varied the relationship between the training and the testing environments. Genotypes were predicted for each environment separately. The data was split into train and test, using the following rules for each of the scenarios:

- Known location/known year (knLoc.knYr): All other environments are included in the training set.
- New location/new year (nLoc.nYr): Environments from the same year or location as the testing environment were excluded.

For all the scenarios, the genotypes to be predicted were excluded from the training set. The determination of ECs for each wheat stage was conducted across all environments before defining the prediction scenario. Thus, the planting date and the dates of wheat stages based on GDD were used even for the ‘nLoc.nYr’ prediction scenario (see below).

For the FW method and JGRA RN, the parameters were estimated in the training set and predicted by genomic prediction for the testing set. For the JGRA marker, the estimation of marker effects was done using environments and genotypes from the training set only.

The within-environment predictive ability was calculated as the Pearson correlation between the observed and the predicted value over the testing set of genotypes and for each environment. Similarly, the across-environment predictive ability is the Pearson correlation calculated when aggregating predicted values across all testing sets, genotypes, and environments.

### Significance of markers from JGRA

For each environment, phenotypic data was scaled to a variance of one and marker effects were estimated using the JGRA marker effect method applied on all genotypes. Then, the marker effects were regressed against the mean phenotype (instead of the environment index determined by the ECs). Finally, the significance of the linear regression of the intercept and slope parameters was estimated. A Manhattan plot was produced from the significance of the intercept and the slope, and the Bonferroni threshold was calculated to account for the multi-testing, such as: $Threshold= -{log}_{10}(\frac{0.05}{m})$, with $m$ the number of markers.

The JGRA reaction norm was also fit using all available genotypes and using the mean phenotype. From the estimation of the intercept and the slope for each genotype, a genome-wide association study (GWAS) was performed using the MM4LMM method [37]. The normality of the residuals was checked with a QQ-plot. The GRM was used to account for the genetic structure. For DIS, 280 individuals were available, and 283 for VSK traits. The same Bonferroni threshold was used for determining significant associations from the Manhattan plot.

## Results

### Phenotyping description

Phenotypic distributions showed variability among the locations and years, indicating a strong impact of the environment. Among the most tested locations, PRP (Prosper, North Dakota) displayed the highest level of FHB, whereas STP (St. Paul, Minnesota) had the lowest level of FHB (Fig. 1C). The correlation between DIS and VSK traits was 0.68. The heritability could not be computed since only entry-mean values were available for a given trial. When extracting BLUPs through a mixed model using the same dataset, Brault et al. [5] obtained reliability values of 0.646 and 0.702 for DIS and VSK, respectively.

The genetic variance accounted for 41.1% and 42.8% of the total variance for DIS and VSK traits, respectively, with slightly less residual variance for DIS (Figure S1). The environmental effect, encompassing location, year, and location-by-year interaction, summed up to 34% and 30% for DIS and VSK, respectively. The GxE component, encompassing the interaction between genotype with location or year, summed up to 5% and 3% for DIS and VSK, respectively (Figure S1).

### Environment description based on environmental covariates

#### Description and comparison of the environment indices

The set of ECs with the highest correlation used only location-specific ECs for VSK with R = 0.49 and V35 for DIS with R = 0.38 (Fig. 2). Adding location variables always increased the accuracy of the model. Interestingly, the location-specific ECs alone had a good correlation for both traits (R = 0.35 for DIS). For the following analysis, we used the best set of ECs for each trait, i.e., V35 for DIS and location-specific ECs alone for VSK.

The weights associated with each EC varied among the traits but the sign of the effects was consistent (Figure S2). The ECs affecting the most DIS trait were TOTC ( +), GWETROOT ( +), N (-), and ECEC (-). Lots of location-specific ECs were among the ECs with the highest absolute value. For the VSK trait, the selected set of ECs included only location-specific ECs, with TOTC ( +), SILT ( +), TCEQ (-), and LON (-) being the most significant variables. Signs of the EC weights were indicated with ( +) and (-) for positive and negative, respectively.

The two environment indices from the PLSR and the sum of three ECs were compared with the JGRA methods. The results showed that the predictive ability values were mostly similar between the two indices, except for JGRA RN, with a superiority of the environment index from PLSR (Figure S3). Therefore, this environment index was kept for the following analyses.

#### Description of the ERM matrix

The ERM showed a mild structure among the locations (Figure S4). As expected, some locations geographically close to each other were more related in the ERM, such as FRG (Fargo, ND, USA) and PRP (Prosper, ND, USA), as displayed in Fig. 1B. Some locations displayed highly differentiated years, such as BRK (Brookings, SD, USA) and CRK (Crookston, MN, USA). Diagonal stripes were visible in the off-diagonal squares (between different locations), indicating higher relatedness of environments from the same year (Figure S4).

### Prediction methods results

#### Finlay-Wilkinson results

- **Within-environment results**

Predictive ability for the intercept parameter was almost twice as high compared to the predictive ability for the slope parameter with mean values of 0.78 and 0.83 for intercept, and 0.37 and 0.47 for slope, for DIS and VSK, respectively (Figure S5A). The predictive ability for predicting phenotypes was 0.46 and 0.50 for the two traits. There was a little effect of the prediction scenario, with a maximum decrease of predictive ability of 0.03.

- **Across-environment results**

Predictive ability across all environments was smaller than in within-environment, with values close to 0.32 for DIS and VSK. There was a small, expected decrease in predictive ability values over the two scenarios of 0.015 for DIS and 0.04 for VSK (Figure S5B).

#### JGRA results

- **Within-environment results**

**Fig. 3:**
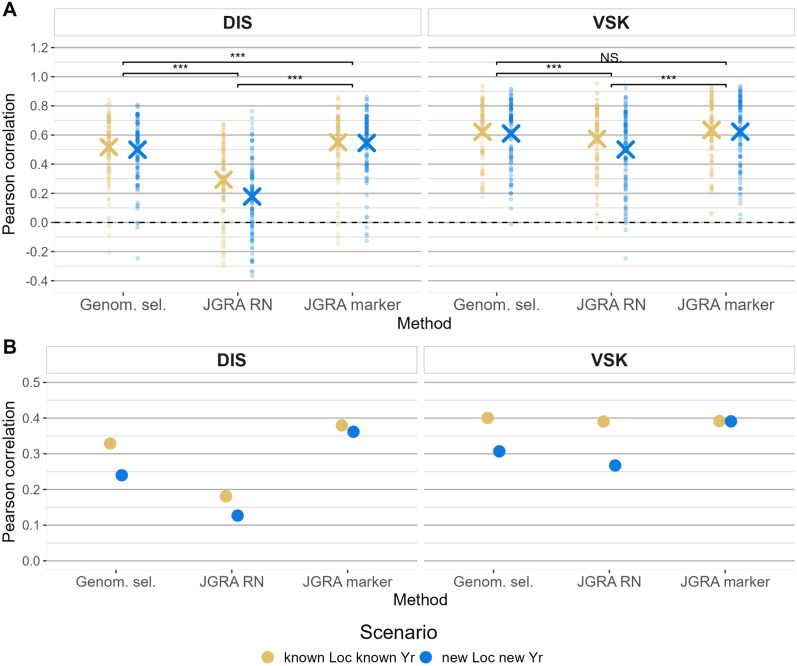
Comparison of predictive ability for JGRA methods. **A**: Within-environment predictive ability for disease index (DIS) and visual scabby kernel (VSK) traits, three methods and two prediction scenarios. Paired *t-tests* were done to compare the significance of the difference of predictive ability between the methods (with the two scenarios), “***” indicates a p-value below 0.001, and “NS.” a p-value above 0.1. The cross represents the mean predictive ability, while the points are the individual predictive ability in each environment.** B**: Across-environment predictive ability for the two traits, three methods, and two prediction scenarios

Predictive ability values within environments were highest for the JGRA marker effect (average of 0.59), and lowest for JGRA RN (average of 0.39), and intermediate for GS (average of 0.56). All the pairwise comparisons between the methods were highly significant (p-value < 0.01) based on a *t-test*, except for the comparison between GS and JGRA marker effect for VSK trait (Fig. 3A). Predictive ability was higher for VSK than for DIS (mean predictive ability of 0.59 and 0.43). The range of predictive ability values was very large for all methods, from -0.30 to 0.86 for JGRA RN for the “knLoc.knYr” scenario and DIS trait, and from − 0.14 to 0.98 for JGRA marker effect for the “knLoc.knYr” scenario and DIS trait.

- **Across-environment results**

The predictive ability across environments was smaller than in within-environment, with a smaller range (Fig. 3B). Otherwise, similar trends were observed, with the same ranking between the methods, traits, and scenarios. For example, for DIS and the “knLoc.knYr” scenario, predictive ability values were 0.380, 0.330, and 0.181 for JGRA marker effect, GS, and JGRA RN, respectively. Interestingly, there was a smaller difference in predictive ability between the two scenarios for the JGRA marker effect than for GS, indicating that the former did comparatively better in the most challenging scenario.

#### Mixed model results

**Fig. 4:**
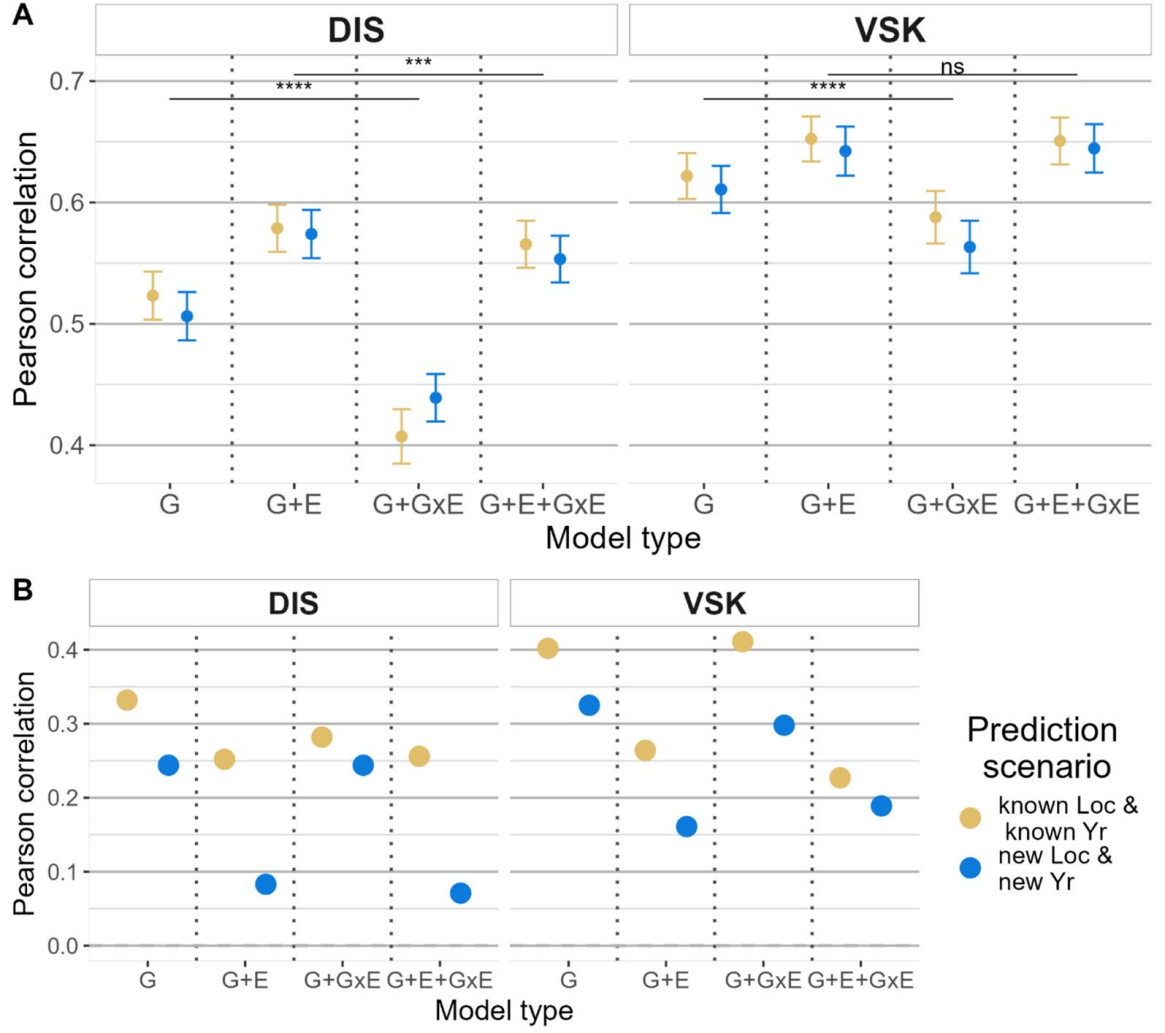
Comparison of predictive ability for mixed models. Mixed model results for the BayesB method for two traits (DIS: disease index and VSK: visual scabby kernel), two prediction scenarios (known location and known year, new location and new year), and four model types. **A**: within-environment predictive ability, **B**: across-environment predictive ability. Paired *t-tests* were conducted to compare the significance of the difference of predictive ability between the methods when including or not including GxE in the model, “***” indicates a p-value below 0.001, “**” indicates a p-value below 0.05, and “*” indicates a p-value below 0.1

Similar trends were observed between the two traits for the mixed model results (Fig. 4). As expected, there was a decrease in the predictive ability between the two scenarios, but the magnitude of decrease was a bit more pronounced in across-environment prediction (Fig. 4B), with a decrease up to 0.185 for DIS and G + E + GxE, compared to a maximum decrease of 0.017 in within-environment prediction for the G model and DIS trait.

Interestingly, models that included the environment effect (i.e., G + E and G + E + GxE) were superior in within-environment, and G + E was the best among those two, with an average predictive ability of 0.576 and 0.647 for DIS and VSK traits, respectively (Fig. 4A). In across-environment, the model types that didn’t include the environment effect (G and G + GxE) were the best, especially for the new location and new year scenario (Fig. 4B).

### Comparison of methods

**Fig. 5:**
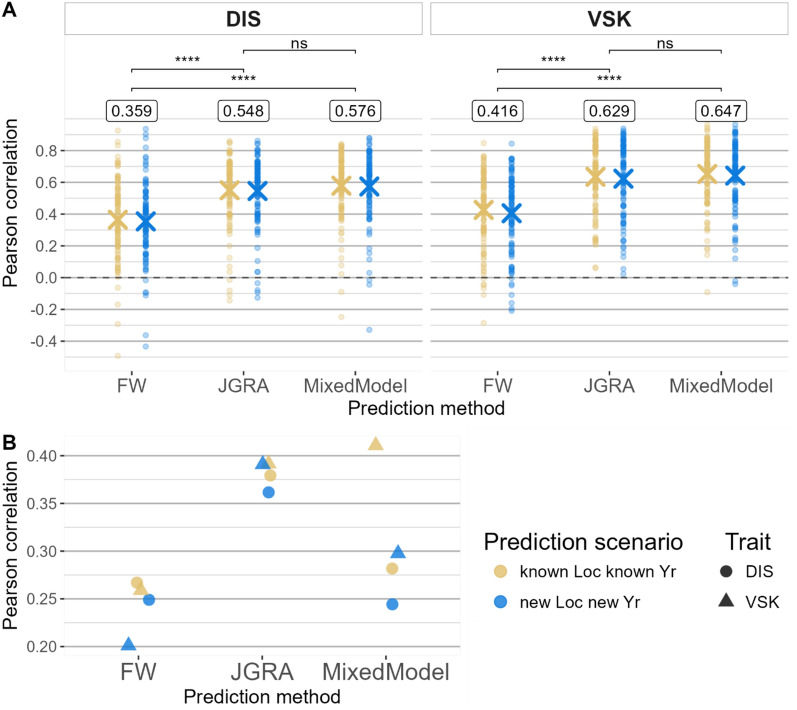
Comparison of predictive ability for FW, JGRA, and mixed models. Comparison of three prediction models: FW: Finlay-Wilkinson regression, using an environment index, JGRA: joint genomic regression analysis, with the marker effect type of model, and mixed model using the BayesB method, and G + E + GxE model type in within-environment (**A**), and G + GxE model type in across-environment (**B**)

For the comparison of methods, the best feature for each method was used, so JGRA marker effect was selected for JGRA. For mixed models, in within-environment, the G + E model was selected, and the G + GxE model was selected in across-environment. Results showed that JGRA and mixed model were the best models in within-environment, with non-significant differences for both traits (Fig. 5A).

The ranking of methods was the same both within- and across-environments, with the lowest predictive ability for FW, followed by mixed models and JGRA (Fig. 5). In within-environment, FW was significantly lower than the other two methods, although there was no significant difference between JGRA and the mixed model. Predictive ability was little affected by the trait or the prediction scenario (Fig. 5A). In across-environment, FW had the lowest predictive ability, followed by mixed models and JGRA. Specifically, there was a stronger drop of predictive ability when predicting new location and year in mixed models, than in JGRA (Fig. 5B). For example, for DIS trait, in across-environment and for new location & new year scenario, predictive ability was of 0.362 and 0.244 for JGRA and mixed models, respectively. For JGRA and mixed models, VSK was slightly better predicted than the DIS trait, although it was the opposite for the FW method.

### JGRA marker significance

**Fig. 6:**
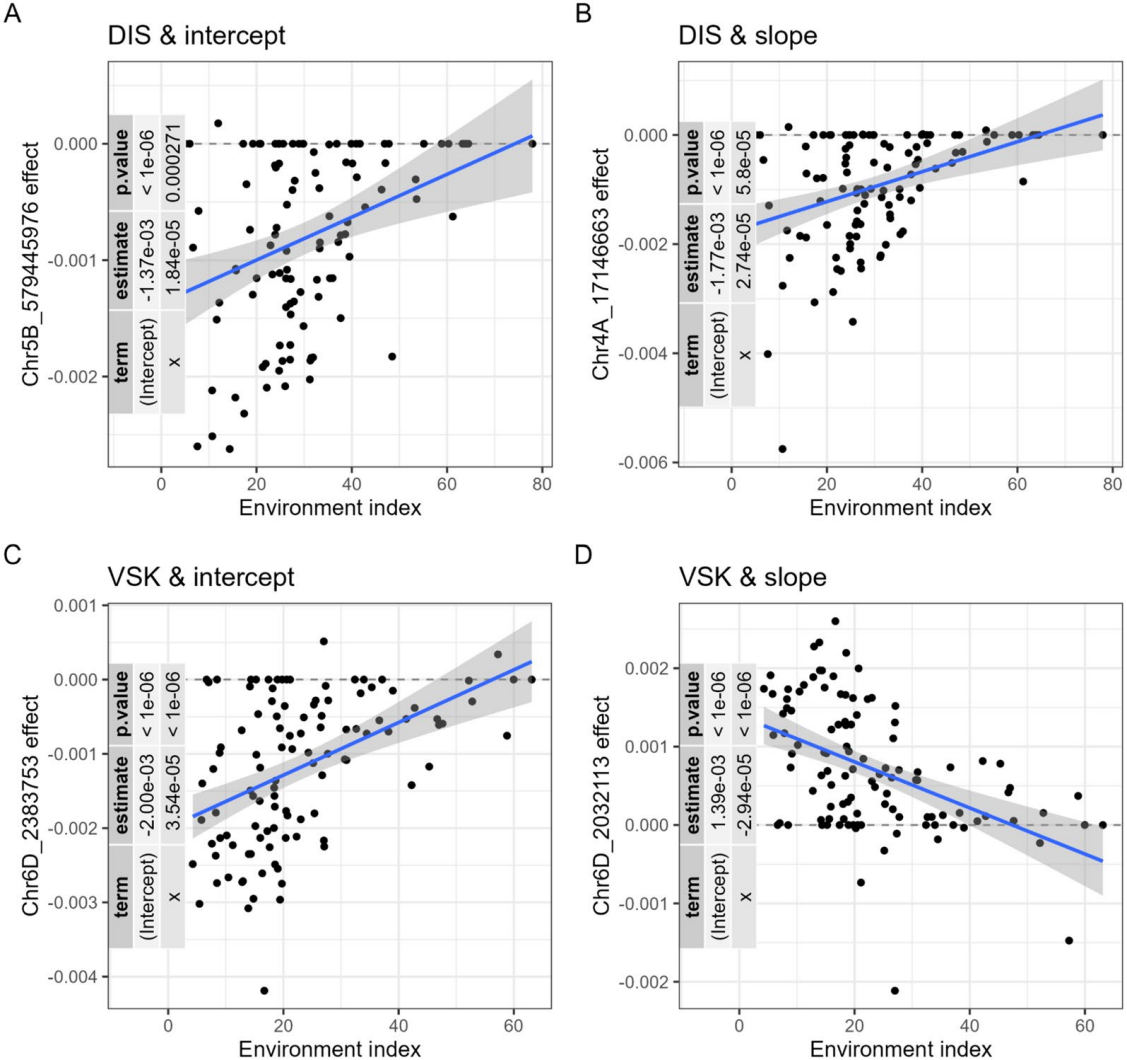
Regression of the marker effects of two traits for the intercept and slope parameters. Regression plot of the most significant markers of intercept (**A**&**C**) and slope (**B**&**D**) parameters for DIS (**A**&**B**) and VSK (**B**&**D**) traits from the JGRA marker effect. The name of the single nucleotide polymorphisms (SNP) with the highest -log_10_ p-value were displayed in each subplot. Each dot represents a marker effect estimated in a different environment. The table on the left of each subplot summarizes the linear regression analysis of the marker effect against the environment index (corresponding here to the mean phenotype). The label of the y-axis represents the marker name with the chromosome number and its physical position

When refitting the JGRA marker effect method, marker effects were regressed against the mean phenotype. From that, many markers were highly significant for the intercept parameters, with -log_10_ p-values up to 19.7 and 12.7 for VSK and DIS, respectively (Fig. 6).

For the slope parameter, the significance levels were smaller.

The most significant SNP for VSK and intercept parameter was located on chromosome 6D, at 2.4 Mbp (megabase pair). The most significant SNP for VSK trait and slope parameter (-log_10_ p-value = 6.73) was located on chromosome 6D at position 2.03 Mbp. These markers were close and for both of them, the intercept and the slope were significant. The most significant SNP for DIS and intercept parameter was located on chromosome 5B, at 579 Mbp (-log_10_ p-value = 12.7). For the slope, the most significant marker was located on chromosome 4A, at 17.1 Mbp (-log_10_ p-value = 4.24) was located on chromosome 6D at position 2.03 Mbp. A significant slope means that the estimated marker effect was more positive (or negative) as the mean phenotype increased (environment with more FHB pressure).

One *Fhb1* QTL (IFA-FM227, located on chromosome 3B, at 13.8 Mbp) included in the genotypic array had a significant effect on VSK and DIS intercepts, with a -log_10_ p-value of 9.21 and 6.39, respectively.

A regular GWAS was applied on the intercept and slope parameters estimated using the JGRA RN method. The results in Figure S6 showed that the significance levels were much lower, with only one marker reaching the Bonferroni significance threshold for VSK and the intercept parameter for a marker located on chromosome 3D at position 352 Mbp (Figure S6C). The ranges of the p-values were roughly the same for both traits and parameters (Figure S6).

### Prediction of best genotypes in each location

**Fig. 7:**
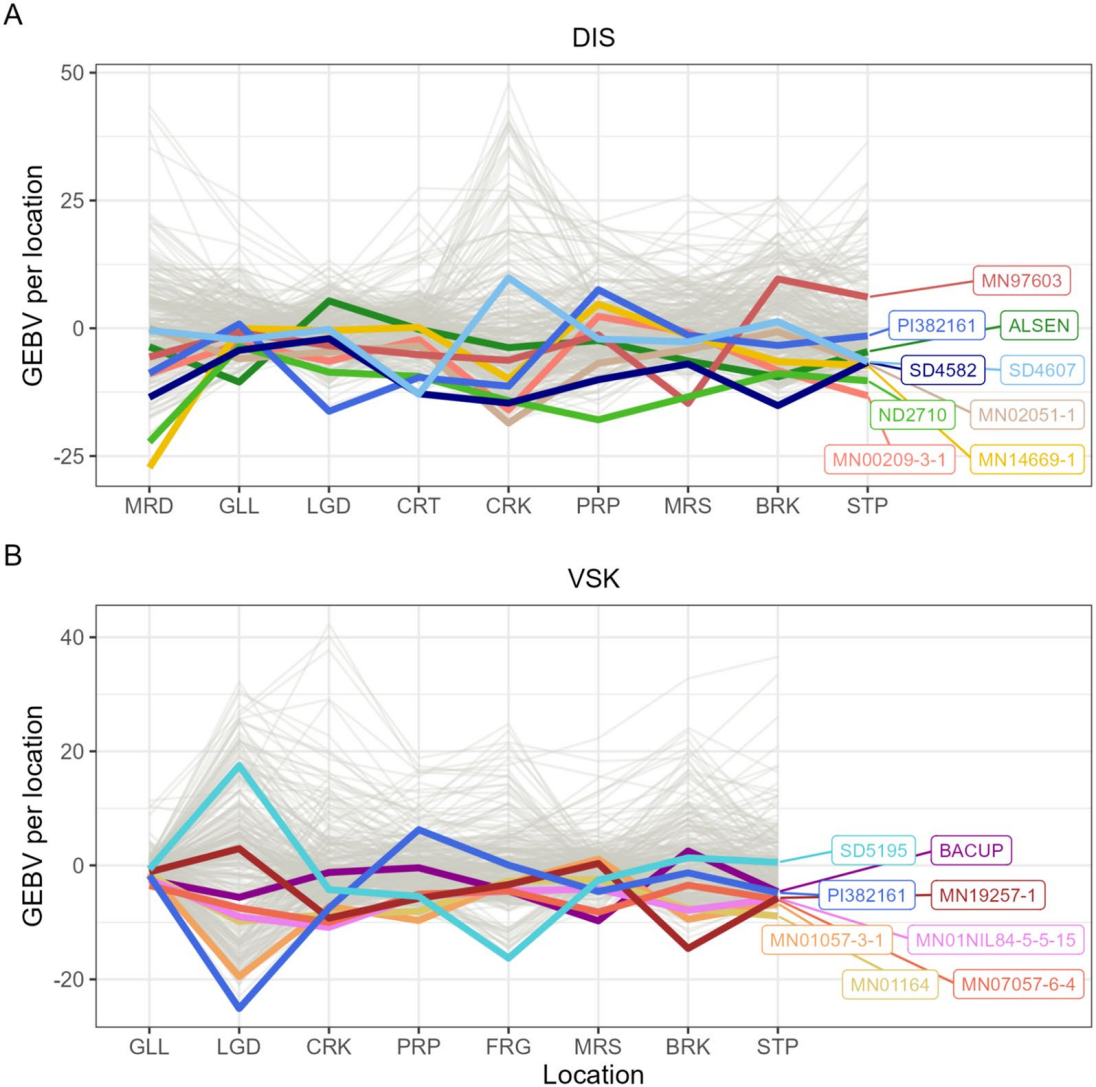
Genomic estimated breeding values (GEBVs) predicted for all genotyped individuals in each location. An environment index was used from a partial least square regression model based on location-specific environmental covariates. The genotypes that were among the best in a given location were highlighted and the genotype names were indicated on the right part of the figure. **A: disease index trait, B:** visual scabby kernel trait.

The JGRA marker effect was fitted using the whole population, and an environment index composed only of location-specific ECs was used to predict the genomic estimated breeding values (GEBVs) for each location specifically (Fig. 7). From that, the breeders can select the most adapted genotypes in each location. In Fig. 7, some genotypes still display a strong variability between the locations. For example, ‘PI382161' was the best genotype for LGD location (Langdon, North Dakota, USA) with the lowest predicted DIS and VSK genotypic value but was not among the 50% best genotypes for PRP location (Prosper, North Dakota, USA) (Fig. 7). Interestingly, ‘ND2710’ and ‘BACUP’ were among the best genotypes both for having low DIS and VSK and were both among the resistant checks in the URSN population. The ‘ND2710’ genotype was released in 1998 and has in its pedigree both ‘SUMAI 3’, which is a Chinese cultivar as a source of the genetic resistance to FHB, and ‘WHEATON’, which is one of the susceptible checks tested in this nursery.

## Discussion

This study provided a comprehensive analysis of the gain brought by ECs in the prediction of G and GxE for two FHB traits. The comparison of three methods, two scenarios, and two traits indicated that the JGRA marker effect was the best method overall and was significantly better than GS, which didn’t incorporate ECs. Predictive ability remained quite stable across the traits and prediction scenarios implemented, suggesting that JGRA was a robust method for a wide range of implementation. Many significant marker-trait associations were found from the output of the JGRA marker effect for both intercept and slope parameters. To our knowledge, these results are the first peer-reviewed attempt to predict GxE based on ECs focusing on a plant disease-related trait.

### Scenarios implemented

In the GxE literature, prediction scenarios are traditionally referred to as CV1 and CV2 for predicting untested and tested genotypes in a tested environment, respectively [9]. Unlike other publications, CV2 is used as our baseline scenario (“KnLoc.knYr”). The scenario of unknown year and location (“nLoc.nYr”) is more challenging because the environments used in the training set are less related to the validation set. Few publications have implemented this type of prediction scenario [22].

Other scenarios could have been compared, such as excluding the location of the testing set environment from the training set or similarly excluding the year. Given the experimental design, these scenarios would yield similar results to the scenarios tested here, because most of the genotypes were tested for only one year. Thus, the base scenario tested “knLoc.knYr” is already excluding almost all observations from the year to be predicted.

Another alternative would have been to include observations from the testing environment in the training set, but its applicability was limited in this dataset because of the small number of observations in each environment. Then, subsampling some individuals in the testing set environment to be included in the training set further reduced the size of the testing set, which was on average 18 individuals.

Predicting unknown genotypes was the common factor of the prediction scenarios tested, as we are seeking methods applicable to breeding programs.

### Incorporation of GxE in the prediction model

#### Relative importance of ECs

The set of ECs with the highest predictability of the mean phenotype was not the one around the heading and anthesis stage, i.e., V67 (Fig. 2). Location-specific environmental covariates alone provided a fair correlation with the mean phenotype for a given environment using PLSR (Fig. 2). This means that unpredictable yearly variations were proportionally less important to determine the level of FHB in a given environment. Thus, the prediction of the genotypic value based on GxE will not depend on the year. Nevertheless, these results were related to the context of inoculated and misted trials, where high precipitation and humidity were ensured. The total level of FHB in a given environment will surely be more variable from year to year in a regular trial. In that sense, this study differs from most of the other GxE studies, focused on yield. Indeed, yield was expected to have much stronger environmental variation and GxE than disease resistance under semi-controlled trials. This was consistent with the variance component analysis, where a small proportion of the variation was due to year or genotype-by-year interaction (Figure S1).

Including only biologically meaningful ECs to constitute the environment index slightly decreased the predictive ability of JGRA RN, mostly for VSK in within-environment and for both traits in across-environment (Figure S3). Besides, the environment index based on the sum of three ECs did not correlate with the mean phenotype (data not shown), yet the predictive ability was close to that when maximizing the predictability of the mean phenotype value through PLSR. Then, the correlation between the environment index and the mean phenotype is not a good indicator of the suitability of a given environment index. Further studies are required to provide more explanation on that.

#### Types of modelling integrating ECs

When using mixed models, the environment and GxE effects were incorporated using a matrix containing all ECs. Conversely, the environment index provided a single synthetic value to represent the environment quality. This latter strategy seems to be more powerful in predicting the relative performance across environments. Although the ECs could have been weighed when computing the W matrix, based on the weights estimated by PLSR when determining the environment index.

Weather data at the heading and anthesis stages can play an important role in the disease spreading. Nevertheless, this effect was minimized by the experimental design, which ensured a rather consistent inoculum for all genotypes, whatever their phenology stage. Besides, neither heading nor anthesis dates were available at the genotype level for most of the environments.

A model has been developed to predict the probability of an FHB epidemics, based on weather data around anthesis to help wheat growers determine if a fungicide application is needed [51, 52]. In this tool, the user selects the level of resistance to FHB of their genotype, but the model doesn’t explicitly model or predict GxE.

### Comparison of the methods

In JGRA RN, estimation of intercept and slope is expected to be less accurate because on average, 4.5 observations were available for a given genotype. However, when combining all intercept and slope estimates across all training set genotypes, it constitutes a good training set size for genomic prediction (between 262 to 264 genotypes depending on the scenario and trait). Then, we expect genomic prediction to be accurate, depending on the heritability of the trait.

In the JGRA marker effect, the first genomic prediction step was based on a few observations (average number of observations per environment is 18.2 and 18.6 for DIS and VSK, respectively). Thus, the prediction of marker effects is expected to be less accurate. However, the number of points for the regression of marker effects against the environment index was 107 and 109 for DIS and VSK, respectively, so this regression is expected to be more accurate. When comparing the environment indices (Figure S3), it was clear that the choice of the environment index had a much larger impact on JGRA RN than on JGRA marker effect, for which the predictive ability remained stable. For JGRA RN, a regression of the phenotype against the environment index is fitted for each genotype. Then, the parameters of this regression were highly affected by the choice of the environment index because there was a small number of environments in which each genotype was tested. On the other hand, for the JGRA marker effect, the environment index was used for the regression of marker effects, with more than 100 observations in our experimental design; this probably made the JGRA marker effect less sensitive to the choice of the environment index. Then, the JGRA marker effect should be preferred if a large number of environments are available in the dataset.

Li et al. [33] observed no difference between the JGRA RN and JGRA marker effect methods, probably because the experimental design was balanced, with all genotypes tested in all environments. This was definitely not the case in our study, which highlights the advantage of each method depending on the experimental design.

For the mixed models results, it was observed that including an environment effect in the model increased the predictive ability in within-environment, i.e., when the correlation was calculated separately for each environment (Fig. 4A). Although it seems counterintuitive, we hypothesize that it is explained by a better fitting of the model in the training set.

### Main takeaway for breeding

Overall, adding GxE in the prediction model consistently improved the predictive ability by a small amount, despite the small percentage of variance explained by GxE (Figure S1). This might be explained by the fact that the trials were inoculated and misted, reducing the impact of the environment. This was consistent with less significant marker effects for the slope parameter than the intercept (Fig. 6). Nevertheless, in the predictions, the ranking of the genotypes varied a lot among the locations (Fig. 7). Indeed, the proportion of variance for GxE does not quantify the amount of GxE for each genotype.

The prediction scenario “nLoc.nYr” included *in season* weather data and information about wheat stages that would not be known at the onset of a growing season. In that sense, the environment was not truly unknown, which could contribute to overestimating predictive ability. That is why the prediction of the best genotypes in each location was done using only location-specific ECs (Fig. 7). An alternative is to fit the genomic prediction model with historical weather data, but it is rarely done in the literature [22, 43].

### Comparison with other studies that tested the usefulness of including GxE

In our study, we showed a significant moderate improvement in predictive ability when including GxE in the prediction model, i.e., the JGRA marker effect was better than GS (Fig. 3), and mixed models that include GxE were significantly better for both traits (Fig. 4). Depending on the study and the method used, the same or different conclusions were obtained in other publications. Most of the studies on GxE were focused on traits such as yield and heading date, which are more prone to show GxE than disease resistance. We could not find any study on disease-related traits comparable to what we have conducted here.

Several studies observed no or minor improvement in predictive ability when including ECs [24, 43, 47]. On the contrary, Costa-Neto, Fritsche-Neto, et al. [11, 12] and 27] observed an increase in predictive ability. Ly et al. [38] computed GxE as a random reaction norm over a few ECs, they observed that the ECs were explaining a significant part of the GxE variance, although the gain in prediction was modest. Piepho and Blancon [46] and Tadese et al. [57] used methodologies comparable to the JGRA RN method. They both found a significant increase in predictive ability when including one or two synthetic covariates.

Compared to previous studies, our across-environment predictive ability was much lower. We hypothesize that it might be due to the lower environmental variability for the type of traits we focused on.

Li et al. [32] was one of the only studies that incorporated GxE to decipher the genetic architecture of the GxE components of trait variation by computing a GWAS on the intercept and slope parameters from the JGRA RN method. They found several already well-known loci for flowering time trait, however, they didn’t uncover new loci by harnessing the marker effects from JGRA, as we did in Fig. 6. The p-values are arising from a linear regression between marker effects and the mean phenotype, thus, they are not comparable to a GWAS. We hypothesized that since marker effects were estimated using the RR-BLUP method, all markers had a non-zero effect, potentially inflating the significance level of the intercept parameter.

This study represents the first attempt to compare three major types of methods for modelling GxE in plant breeding. For the first time, disease resistance traits were predicted using ECs, in challenging prediction scenarios in which both the genotype and the environment were untested.

## Supplementary Information

Supplementary material 1.

## Data Availability

Phenotypic data from all URSN trials are available on the T3 database (https://wheat.triticeaetoolbox.org/). All scripts, intermediate files, result tables, and figures to reproduce the results are available in a public repository: 10.5061/dryad.wstqjq2z0.
