# Supplementary material for: Improving genomic prediction for plant disease using environmental covariates
Source: Plant Methods. 2025 Aug 20;21:114. doi: 10.1186/s13007-025-01418-0 (PMC12366029; doi:10.1186/s13007-025-01418-0)
Supplement: Supplementary file 1 — Supplementary material 1. [file 13007_2025_1418_MOESM1_ESM.pdf]

Improving Genomic Prediction For Plant Disease Using Environmental Covariates

Supplemental Material

Charlotte Brault[1,*], Emily J. Conley[1], Andrew J. Green[2], Karl D. Glover[3], Jason P. Cook[4], Harsimardeep S. Gill[1], Andrew C. Read[5], Jason D. Fiedler[6,*], and James A. Anderson [1]

[1]Department of Agronomy and Plant Genetics, University of Minnesota, Saint Paul, Minnesota, USA

[2]Department of Plant Sciences, North Dakota State University, Fargo, North Dakota, USA

[3]Agronomy, Horticulture, and Plant Science Department, South Dakota State University, Brookings, South Dakota, USA

[4]Plant Sciences and Plant Pathology Department, Montana State University, Bozeman, Montana 59717, USA

[5]USDA-ARS, Plant Science Research Unit, St. Paul, Minnesota, USA

[6]USDA-ARS Cereal Crops Improvement Research Unit, Edward T. Schafer Agricultural Research Center, Fargo, North Dakota, USA

[*]Corresponding authors: charlotte.brault@live.com; jason.fiedler@usda.gov

List of Figures

Figure S1 Variance component analysis 4
Figure S2 PLSR coefficients from environmental covariates 5
Figure S3 Comparison of environment indices for JGRA prediction 6
Figure S4 Environmental relationship matrix 7
Figure S5 Finlay-Wilkinson results 8
Figure S6 Manhattan plot from JGRA RN 9

List of Tables

Table S1 Weather station ID 3

Table S1: NOAA weather station ID for uniform regional scab nursery (URSN) location trials.

Country	State	Location	LocationCode	WeatherStationID
USA	Minnesota	Crookston	CRK	USC00211891
USA	Minnesota	St. Paul	STP	USC00321362
USA	Minnesota	Morris	MRS	USC00215638
USA	Montana	Bozeman	BZM	USC00241044
USA	Montana	Sidney	SDN	USC00247560
USA	North Dakota	Minot	MNT	USC00325993
USA	North Dakota	Carrington	CRT	USC00321362
USA	North Dakota	Casselton	CST	USC00321408
USA	North Dakota	Hettinger	HTG	USC00324180
USA	North Dakota	Fargo	FRG	USW00014914
USA	North Dakota	Williston	WLT	USC00329430
USA	North Dakota	Forman	FRM	USC00323117
USA	North Dakota	Langdon	LGD	USC00324958
USA	North Dakota	Thompson	TPS	USC00323621
USA	North Dakota	Prosper	PRP	USW00014914
USA	South Dakota	Brookings	BRK	USC00391076
USA	South Dakota	Selby	SLB	USC00397545
USA	South Dakota	Groton	GRT	USW00014929
USA	Wyoming	Powell	PWL	USC00487388
USA	Washington	Pullman	PLM	USW00094129
CAN	Manitoba	Morden	MRD	CA005021849
CAN	Manitoba	Winnipeg	WNP	CA00502S001
CAN	Manitoba	Brandon	BRD	CA005010490
CAN	Manitoba	Glenlea	GLL	CA00502S001
CAN	Saskatchewan	Swift Current	SWC	CA004028060

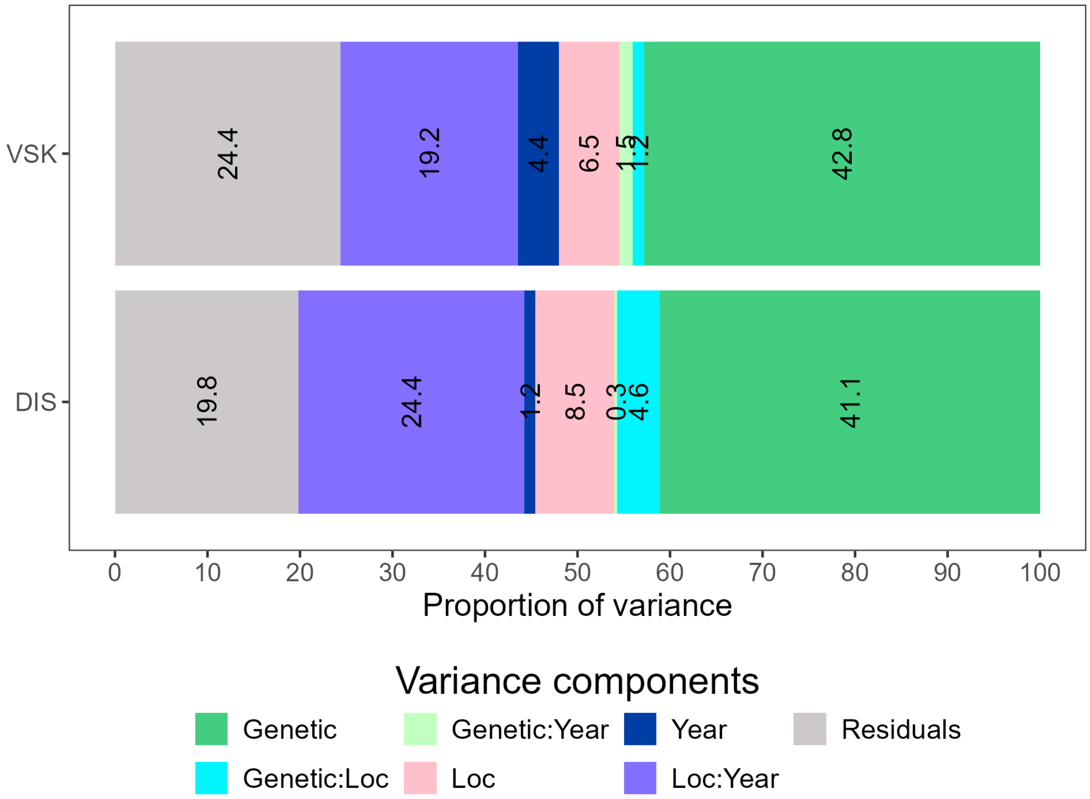

Figure S1: Variance components for visual scabby kernel (VSK) and disease index (DIS) traits, obtained with a mixed model fitted on genotypes replicated more than one year. ":" corresponds to an interaction, Loc: location effect. Numbers indicate the proportion of variance explained for each component.

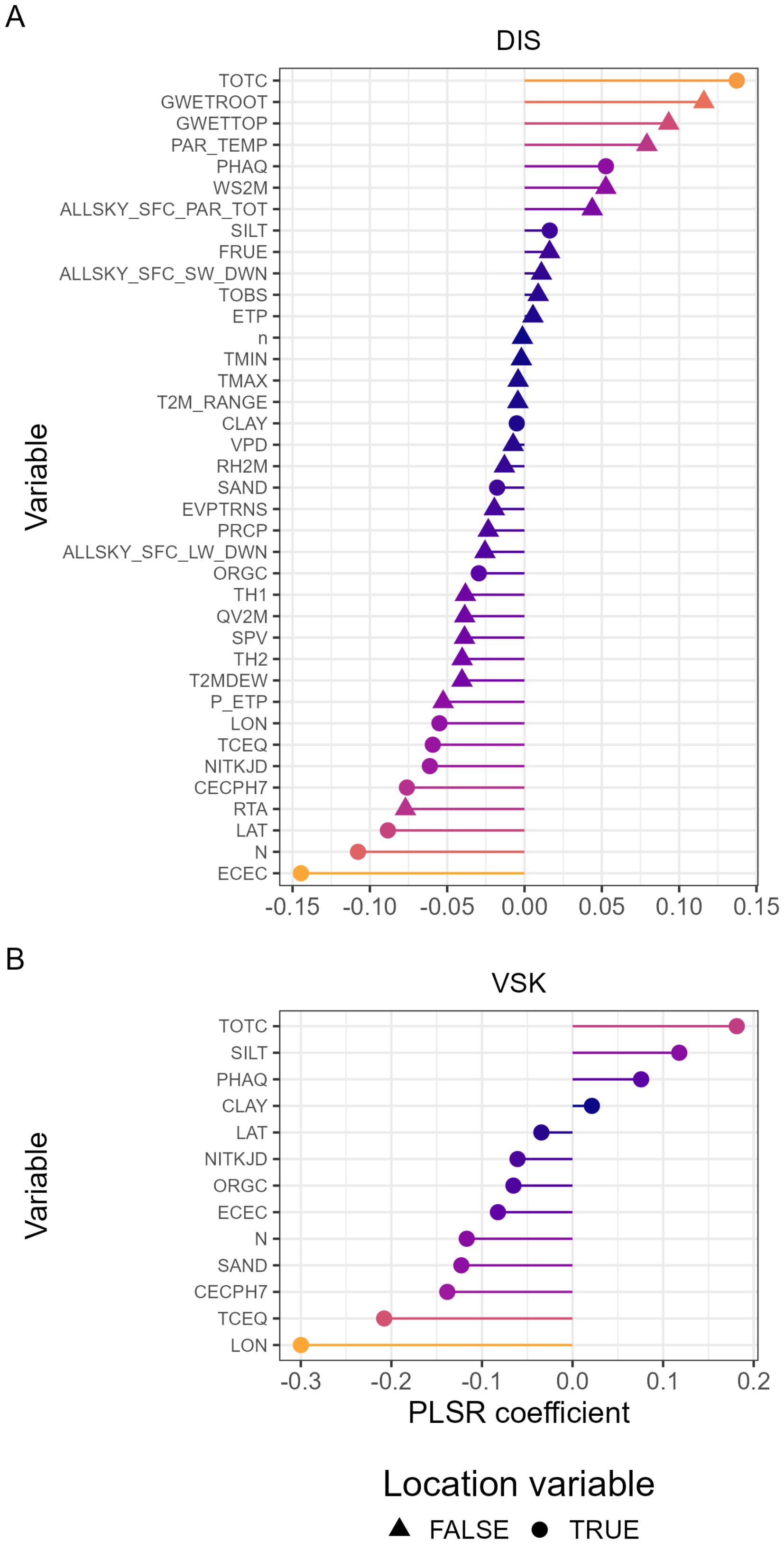

Figure S2: Partial least square regression (PLSR) coefficients associated with mean phenotype prediction for DIS (A) and VSK (B) traits for the most predictive wheat set of environmental covariates (i.e., stage sowing to double ridge for DIS and with soil variables and only soil variables for VSK trait). The point shape indicates if the environment covariate varies on a daily basis, or if it varies on a location basis. Variable description is available in Table 1.

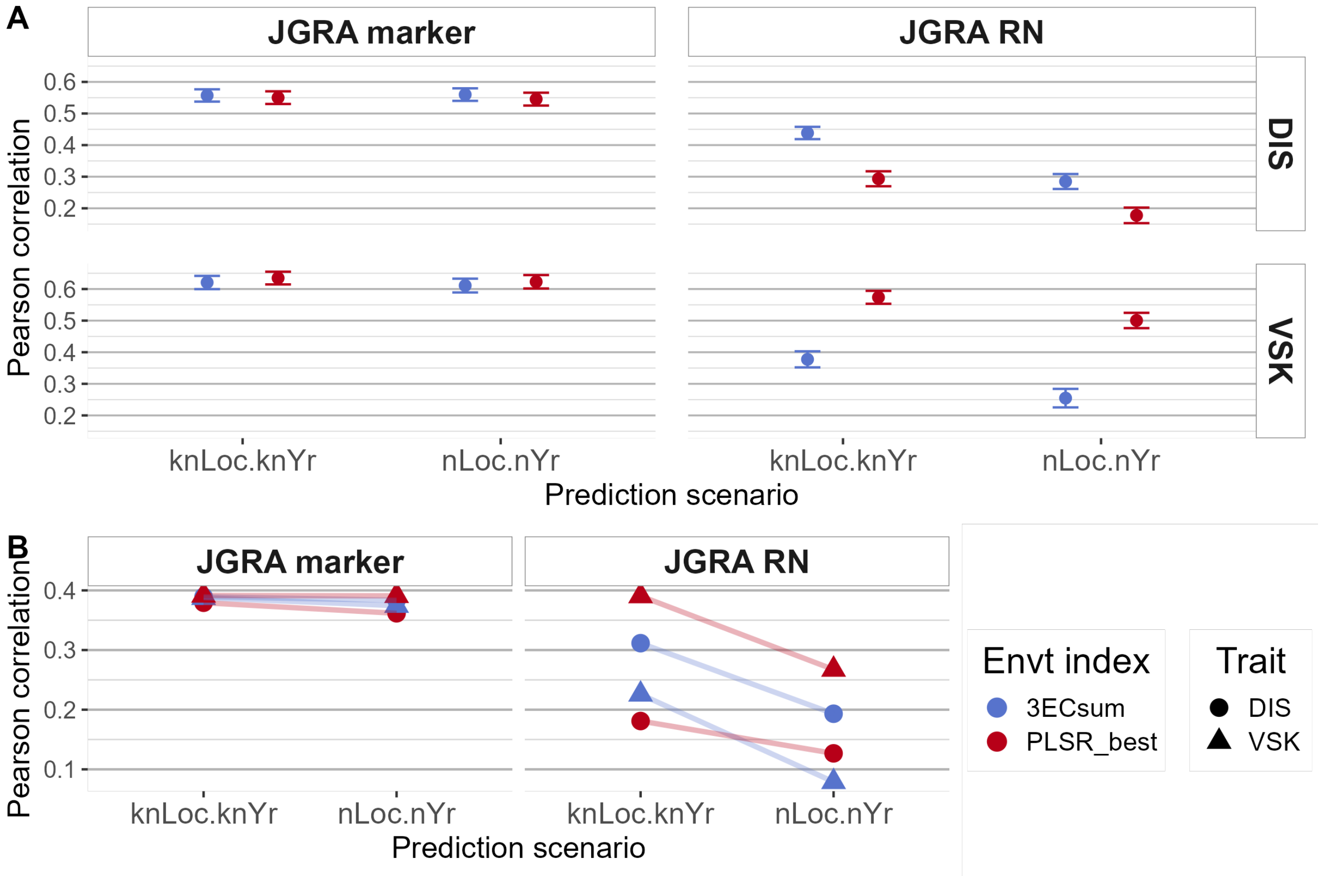

Figure S3: The environment index 3EC sum corresponds to the sum of 3 environmental covariates (precipitation, temperature, and relative humidity), averaged between 14 days around anthesis. The other environment index was composed of all ECs from the best set selected previously. A: within-environment predictive ability, B: across-environment predictive ability.

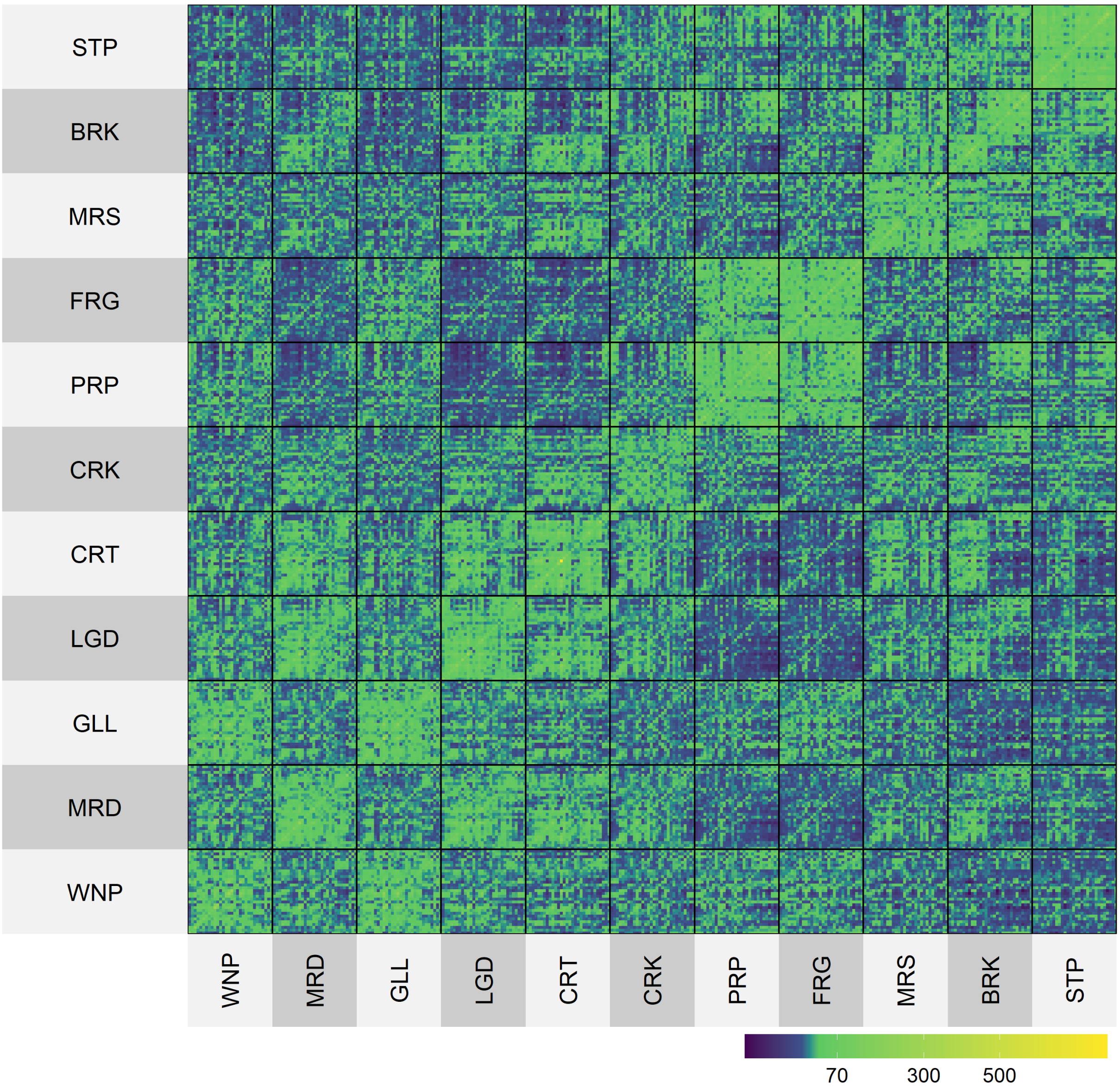

Figure S4: Environmental relationship matrix (ERM), computed from environmental covariates harnessed over the 1995-2024 period. Each pixel represents an environment (a year-location combination), each square represents a location. The color represents the relatedness between the environments from low (dark blue) to high (yellow).

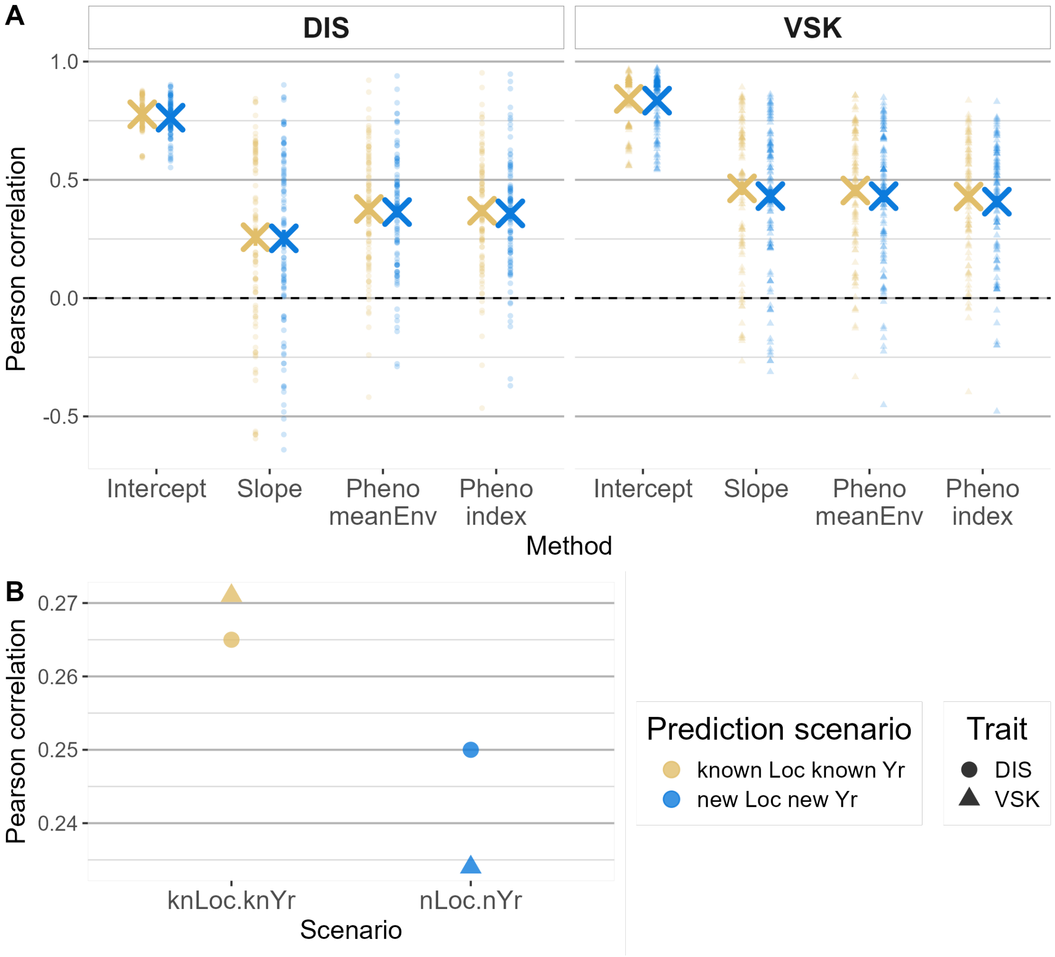

Figure S5: Finlay-Wilkinson (FW) results for DIS and VSK traits and two prediction scenarios, A: predictive ability calculated within-environment. Comparison of predictive ability for intercept and slope parameters, and for prediction of phenotype based on the mean phenotype as an environment index (Pheno meanEnv) or based on the environment index from environmental covariates (Pheno index). B: predictive ability (using Pheno index) calculated across all environments.

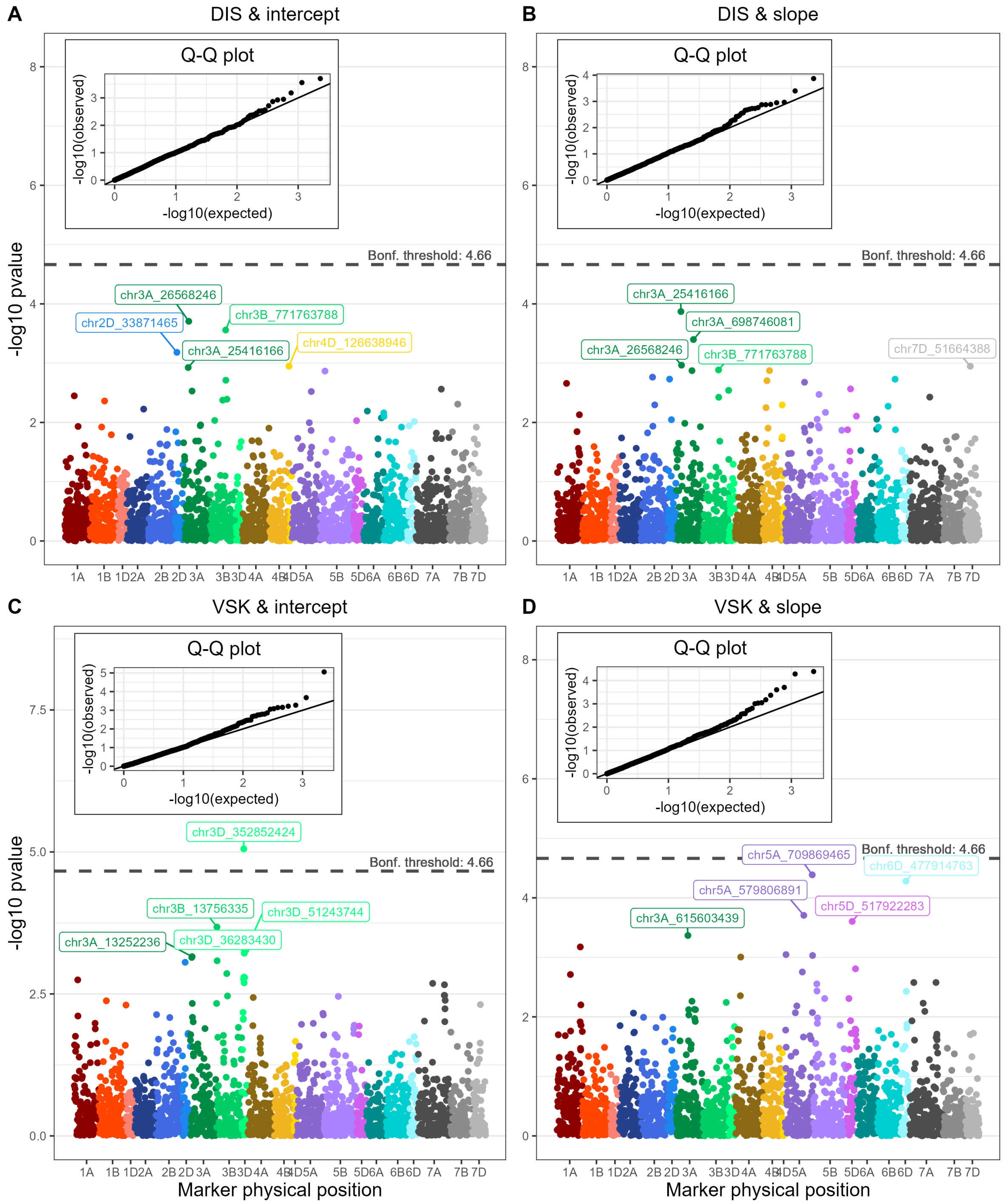

Figure S6: Manhattan plot from genome-wide association study (GWAS) analysis based on joint genomic regression analysis reaction norm (JGRA RN) estimates of slope and intercept parameters. A & B: for disease index (DIS) trait, C & D: for visual scabby kernel (VSK) trait, A & C: for intercept parameter, B & D: for slope parameter. The five most significant SNPs were pinpointed. Each dot represents a marker and its p-value, on a -log10 scale, the color represents the chromosome and markers were ordered by their physical position.
